# Association of serum Interleukin-6 with dysregulated lipid metabolism and nutritional status in patients with pulmonary tuberculosis: a case-control study

**DOI:** 10.1186/s12879-026-12823-8

**Published:** 2026-02-13

**Authors:** Xiaohua Ma, Sifang Xiao, Aichun Tan, Aifeng Liu, Jian Xiao

**Affiliations:** 1 Department of Laboratory, The Affiliated Changsha Central Hospital, Hengyang Medical School, University of South China, Changsha, Hunan 410004 China; 2 Department of Pathology, The Affiliated Changsha Central Hospital, Hengyang Medical School, University of South China, Changsha, Hunan 410004 China; 3 The Pathogenic Microorganism Detection Technology Innovation Center of Changsha, Changsha, Hunan 410004 China

**Keywords:** Pulmonary tuberculosis, Interleukin-6 (IL-6), Lipid metabolism, Nutritional status, Inflammatory cytokines

## Abstract

**Background:**

This study explores correlations between serum IL-6 levels, lipid metabolism, and nutritional status in pulmonary tuberculosis patients to advocate for integrating metabolic-nutritional assessments into routine TB care. It addresses a critical gap in current guidelines by linking IL-6-mediated inflammation to metabolic health, aligning with WHO’s holistic care principles.

**Methods:**

A case-control study was employed, which included 724 hospitalized patients diagnosed with pulmonary tuberculosis (the case group) from the tuberculosis department of a tertiary hospital between January 2023 and December 2023, matched with 724 healthy individuals who served as the control group. The indicators measured included lipid profile [Triglycerides (TG), Total Cholesterol (TC), High-Density Lipoprotein Cholesterol (HDL-C) and Low-Density Lipoprotein Cholesterol (LDL-C)], nutritional indicators [Total Protein (TP), Albumin (ALB) and Globulin (GLB)], and inflammatory indicators (IL-6). Statistical analyses employed SPSS 18.0, utilizing the Mann-Whitney U test for intergroup comparisons and Spearman’s correlation for association analyses, and multiple linear regression to identify independent predictors of serum IL-6 levels.

**Results:**

Compared to the control group, the case group had significantly lower levels of TG, TC, HDL-C, LDL-C, TP, and ALB (*Z=-25.107~-3.619, all P = 0.000), while GLB levels were significantly elevated (Z=-7.113, P = 0.000*). After adjusting for 5 confounders, Spearman’s partial rank correlation analysis showed that TP had a positive correlation with TC, HDL-C and LDL-C (*r = 0.115 ~ 0.181, P = 0.000 ~ 0.002*), ALB had a positive correlation with TC and HDL-C (*r = 0.271 ~ 0.466; all P = 0.000*), while GLB had a negative correlation with HDL-C and LDL-C (*r = -0.096~-0.266, P = 0.000 ~ 0.010*). IL-6 was negatively correlated with TC, TG, HDL-C and LDL-C (*r = -0.971~-0.220, all P = 0.000*), and negatively correlated with TP and ALB (*r = -0.253~-0.163, all P = 0.000*). Multiple linear regression analysis, after controlling for confounders and addressing multicollinearity, further identified lower levels of LDL-C, HDL-C, triglycerides (TG), and globulin (GLB) as significant independent predictors of higher serum IL-6 concentrations (all P < 0.05). The final model explained a substantial proportion of the variance in IL-6 levels (Adjusted R² = 0.913).

**Conclusion:**

This study reveals that patients with pulmonary tuberculosis exhibit a metabolic-nutritional phenotype characterized by “hypolipidemia, hypoalbuminemia, and hyperglobulinemia”. Beyond correlation, multiple linear regression identified specific lipid fractions (LDL-C, HDL-C, TG) and globulin as independent predictors of IL-6 levels, indicating that IL-6 levels are significantly associated with these abnormalities and may reflect a shared underlying dysregulatory pathway. This research reinforces the management concept of viewing tuberculosis as a systemic metabolic disease and highly recommends that clinicians incorporate assessments of specific lipid profiles and protein fractions as a routine component of comprehensive tuberculosis management, with the goal of breaking the vicious cycle of “tuberculosis, malnutrition, and decreased immunity.” This approach could ultimately lead to better treatment outcomes for tuberculosis worldwide.

**Clinical trial number:**

Not applicable.

**Supplementary Information:**

The online version contains supplementary material available at 10.1186/s12879-026-12823-8.

## Introduction

Tuberculosis (TB) remains one of the deadliest infectious diseases in the world, posing a persistent and severe challenge to public health. According to the latest report from the World Health Organization (WHO), about 10.8 million new TB cases were reported worldwide in 2023, resulting in 1.25 million deaths [1]. Despite progress in diagnosis and treatment, managing tuberculosis, especially its complex complications and systemic effects, is still a major challenge. Lung tuberculosis is not just a pulmonary infectious disease; it is a syndrome accompanied by systemic inflammatory responses, metabolic disorders, and nutritional depletion, all of which contribute to poor patient prognosis, poor treatment tolerance, and increased risk of epidemic transmission [2, 3].

For a long time, malnutrition has been considered an important risk factor and consequence of tuberculosis, which creates a vicious cycle [4]. Increasing evidence suggests that patients with tuberculosis often have dyslipidemia and hypoalbuminemia, and these metabolic changes are closely related to the severity of the disease and clinical outcomes [5, 6]. This “wasting” phenotype indicates that tuberculosis pathogenesis extends beyond direct host-pathogen interaction and might include a broader “inflammation-metabolism-nutrition” triad driven by chronic inflammation [7].

In this triad, cytokines are key messengers. Interleukin-6 (IL-6), as a multifunctional pro-inflammatory cytokine, is significantly higher during the acute phase and immune response of tuberculosis [8]. It is known that IL-6 can inhibit the expression of key enzymes involved in lipid synthesis, promote the production of acute phase proteins in the liver, and lead to muscle protein breakdown, thereby participating in the development of cachexia [9]. However, how IL-6 mediates dyslipidemia and worsens nutritional status in tuberculosis is still not well understood. The connections and mechanisms involved have not been systematically explained.

Therefore, this study aims to identify how lipid levels, nutritional status, and inflammatory indicators change in patients with lung tuberculosis through a case-control study, focusing on the correlation between IL-6 levels and lipid metabolism as well as nutritional status, to uncover how IL-6 might work in the “inflammation-metabolism-nutrition” triad of tuberculosis. The research findings will provide a scientific basis for optimizing comprehensive management strategies for tuberculosis, highlighting the need for regular assessment and intervention in metabolic and nutritional status along with anti-tuberculosis treatment.

## Methods

### Patients and methods

A case-control design was used, which included 724 hospitalized patients diagnosed with pulmonary tuberculosis from January to December 2023 (case group), matched with 724 healthy individuals getting check-ups during the same period (control group). All cases of pulmonary tuberculosis were diagnosed based on the “WS 288–2017 Tuberculosis Diagnosis Criteria” relying on clinical signs, imaging results, and positive lab tests, including any positive results from tuberculosis cultures, sputum smears, or molecular tests for Mycobacterium tuberculosis. Inclusion criteria: ① Age 16 or older; ② Confirmed pulmonary tuberculosis. Exclusion criteria: ① HIV/AIDS- infected individuals; ② History of cancer; ③ Severe heart failure; ④ Active viral hepatitis or other infectious diseases; ⑤ Pregnant women. Healthy Control Inclusion criteria: Age/gender-matched institutional exam participants with no recent infections, malignancies, severe organ dysfunction (e.g., hepatic), or psychiatric/cognitive disorders, Exclusion criteria: Malignancies, HIV/AIDS-infected individuals and pregnant women. We used a double-blinded data entry system to handle case information, anonymizing sensitive fields such as names, contact information, and ID numbers.

### Testing indicators

After fasting for 8 h, we collected 5 mL of blood from all subjects, and serum was obtained after centrifugation for testing. We measured:

1. **Lipid Profile**: Triglycerides (TG), Total Cholesterol (TC), High-Density Lipoprotein Cholesterol (HDL-C), Low-Density Lipoprotein Cholesterol (LDL-C), using kits from Meikang Biotechnology, and tested with a Hitachi 7600 biochemical analyzer.
2. **Nutritional Indicators**: Total Protein (TP), Albumin (ALB), using kits from Meikang Biotechnology; Globulin (GLB) was calculated (GLB = TP - ALB).
3. **Inflammatory Indicators**: IL-6 (Electrochemiluminescence method), using kits from Beijing Hotgen Biotech Co., Ltd., and tested with a C2000 chemiluminescence analyzer.

We conducted all tests with internal quality control and passed the inter-laboratory quality evaluation by the National Health and Family Planning Commission Clinical Laboratory Center (annual pass rate 100%).

### Construction and variable selection of multiple linear regression models

To identify independent predictors of serum IL-6 levels, hierarchical multiple linear regression was performed after confirming non-normal data distribution. Potential multicollinearity among lipid and nutritional indicators was rigorously addressed. Variable configurations were systematically compared based on statistical significance of predictors (P < 0.05), variance inflation factors (VIF < 5 considered acceptable), and clinical interpretability. Consequently, TC was excluded due to severe collinearity masking individual lipid effects, and LDL-C, HDL-C, and TG were retained. For nutritional indicators, only the combination of TP and GLB yielded a significant independent predictor (GLB). The final model thus included LDL-C, HDL-C, TG, TP, GLB, along with demographic and clinical covariates (Supplementary Table S1 for the selection process). Results are presented as unstandardized (B) and standardized (β) coefficients with 95% confidence intervals (CI).

### Statistical methods

We used SPSS 18.0 for statistical analysis. The normality of continuous variables was assessed separately for the case and control groups using the Shapiro-Wilk test. Normally distributed measurement data were expressed as mean ±standard deviation, and inter-group comparisons were conducted using independent samples t-test; non-normally distributed data were expressed as median (interquartile range), and inter-group comparisons were conducted using Mann-Whitney U test. Bivariate correlation analysis and Spearman’s partial rank correlation analysis were performed based on data distribution. Multiple linear regression was used to identify independent predictors of serum IL-6 levels.Exact p-values are reported throughout the manuscript, and we considered a p-value less than 0.05 to be statistically significant.

## Results

### Clinical characteristics of tuberculosis patients

Demographic characteristics of the case group revealed that males comprised 66.6% (482/724) with a male-to-female ratio of 1.99:1 and a median age of 58 years (IQR: 47–68 years). Among these patients, 99.4% (700/724) were treatment-naïve, 42.1% (305/724) were sputum smear-positive, 21.8% (158/724) had a history of diabetes, 20.6% (149/724) had hypertension, and 9.8% (71/724) had chronic kidney disease (CKD). In the control group, males comprised 67.0% (485/724) with a median age of 57 years (IQR: 45–68 years). Comparisons between the tuberculosis and control groups revealed no statistically significant differences in gender distribution (*χ²=0.022, P = 0.882*) or age (*Z=-0.713, P = 0.476*), minimizing confounding factors in this population (Table 1).

**Table 1:** Comparative analysis of baseline characteristics between groups

Group	Male (*n*, %)	Age (year)*	diabetes	hypertension	CKD	Treatment -naive	Smear -positive
Control group (*n* = 724)	485 (67.0%)	57 (45,68)	/	/	/	/	/
Case group (*n* = 724)	482 (66.6%)	58 [47,68]	158	149	71	700	305
*χ²/Z*	0.022	-0.713	/	/	/	/	/
*P-value*	0.882	0.476	/	/	/	/	/

*Notes: Age is expressed using interquartile range [M (Q1, Q3)] and analyzed using Mann Whitney U test. statistical significance was set at P < 0.05, CKD: chronic kidney disease; IQR: interquartile range

### Metabolic and nutritional characteristics of tuberculosis patients

as illustrated in Table 2, the tuberculosis group exhibited significantly lower levels of TG, TC, HDL-C, LDL-C, TP, and ALB than the control group, while GLB level was significantly higher in the tuberculosis group compared to the control group. Specifically, TC decreased by 12.4% (4.11 vs. 4.69 mmol/L), HDL-C decreased by 17.6% (0.98 vs. 1.19 mmol/L), LDL-C decreased by 13.1% (2.38 vs. 2.74 mmol/L), ALB decreased by 13.6% (38.0 vs. 44.0 g/L), and GLB increased by 7.7% (28.00 vs. 26.00 g/L). all metabolic indicators showed statistically significant differences between the tuberculosis and control groups (*P < 0.001*).

**Table 2:** Comparative analysis of metabolic and nutritional parameters between two groups

Indicators	Control group (*n* = 724)	tuberculosis group (*n* = 724)	*Z*	*P*
TC (mmol/L)	4.69 [4.11,5.31]	4.11 [3.47,4.76]	-11.562	0.000
TG (mmol/L)	1.47 [1.06,2.14]	1.35 [0.99,1.91]	-3.619	0.000
HDL-C (mmol/L)	1.19 [1.01,1.37]	0.98 [0.81,1.23]	-11.923	0.000
LDL-C (mmol/L)	2.74 [2.22,3.27]	2.38 [1.85,2.90]	-8.227	0.000
TP (g/L)	70.00 [68.0,73.0]	65.00 [61.00,69.00]	-17.278	0.000
ALB (g/L)	44.00 [42.0,46.0]	38.00 [33.00,41.00]	-25.107	0.000
GLB (g/L)	26.00 [24.0,28.0]	28.00 [24.00,32.00]	-7.113	0.000

Notes: (1) Data are presented as median (interquartile range). (2) Group comparisons were performed using the Mann-Whitney U test. (3) statistical significance was set at P < 0.05. Indicator is expressed using interquartile range [M (Q1, Q3)]

Abbreviations: TC, Total Cholesterol; TG, Triglycerides; HDL-C, High-Density Lipoprotein Cholesterol; LDL-C, Low-Density Lipoprotein Cholesterol; TP, Total Protein; ALB, Albumin; GLB, Globulin

### Correlation between blood lipids and nutritional indicators in tuberculosis patients

Before and after controlling for confounding factors, the significance of the relationship between TP and the four blood lipid indicators remained consistent, as did the significance of the relationship between ALB and TC/HDL-C. Correlation coefficients between ALB and TC/HDL-C(*r = 0.410 ~ 0.504, all P = 0.000*), were significantly higher than those between TP and TC/HDL-C (*r = 0.139 ~ 0.181, all P = 0.000*). Moreover, after adjusting for five confounders (gender, age, hypertension, diabetes mellitus, CKD), Spearman’s partial rank correlation analysis (for non-normal data) revealed the following changes: ① The significant correlation between GLB and TC became non-significant (pre-adjustment: *r* =-0.106,* P = 0.004*; post-adjustment: *r* =-0.032,* P = 0.389*); ② The non-significant correlation between GLB and LDL-C turned significant (pre-adjustment: *r* =-0.035, P = 0.341; post-adjustment: *r* =-0.096, P = 0.010); ③The significant correlation between ALB and LDL-C became non-significant (pre-adjustment: *r* = 0.279,* P = 0.000*; post-adjustment: *r* = 0.038,* P = 0.309*); ④ TG showed no significant correlation with TP, ALB and GLB after adjustment (all P ≥ 0.05) (as illustrated in Table 3). These results underscore the necessity of adjusting for key demographic and comorbid confounders when exploring lipid-nutrient interactions in TB patients, ensuring the validity of inferred associations and providing a more reliable basis for subsequent mechanistic research.

**Table 3:** Unadjusted and adjusted correlations between lipid metabolic and nutritional parameters (*n* = 724)

Nutrient	TC	TG	HDL-C	LDL-C
TP				
Unadjusted	0.288^***^	0.090^*^	0.199^***^	0.223^***^
Adjusted **†**	0.181^***^	0.037	0.139^***^	0.115^**^
ALB				
Unadjusted	0.410^***^	0.139^***^	0.504^***^	0.279^***^
Adjusted **†**	0.271^***^	0.057	0.466^***^	0.038
GLB				
Unadjusted	-0.106^**^	-0.031	-0.273^***^	-0.035
Adjusted **†**	-0.032	-0.011	-0.266^***^	-0.096^*^

Notes: **†** Adjusted for gender, age, hypertension, diabetes mellitus, and chronic kidney disease (CKD). Data are Spearman correlation coefficients (unadjusted) or partial Spearman correlation coefficients (adjusted). Significance levels: ***, P < 0.001, **, P < 0.01, *, P < 0.05

Abbreviations: TC, Total Cholesterol; TG, Triglycerides; HDL-C, High-Density Lipoprotein Cholesterol; LDL-C, Low-Density Lipoprotein Cholesterol; TP, Total Protein; ALB, Albumin; GLB, Globulin

### Correlation between IL-6 and blood lipid indicators in tuberculosis patients

Spearman’s partial rank correlation analysis (non-normal data) was performed to explore the correlations between IL-6 and lipid indicators, both before and after adjusting for five confounders (gender, age, hypertension, diabetes mellitus and CKD). The results showed that after adjustment, IL-6 remained significantly negative correlated with TC (pre-adjustment: *r* =-0.280,* P = 0.000*; post-adjustment: *r* =-0.971,* P = 0.000*), TG (pre-adjustment: *r* =-0.128,* P = 0.000*; post-adjustment: *r* =-0.220,* P = 0.000*), HDL-C (pre-adjustment: *r* =-0.319,* P = 0.000*; post-adjustment: *r* =-0.270,* P = 0.000*), and LDL-C (pre-adjustment: *r* =-0.200,* P = 0.000*; post-adjustment: *r* =-0.720,* P = 0.000*). No significant fluctuation was observed in the absolute values of partial correlation coefficients, indicating that the above correlations were not significantly affected by the 5 confounders (as shown in Table 4).

**Table 4:** Correlation between inflammatory marker IL-6 and lipid profiles before and after adjustment for confounders (*n* = 724)

Lipid Profile	Unadjusted Correlation with IL-6 (*r*)	Adjusted Correlation with IL-6 (*r*) †
TC	–0.280^***^	–0.971^***^
TG	–0.128^***^	–0.220^***^
HDL-C	–0.319^***^	–0.270^***^
LDL-C	–0.200^***^	–0.720^***^

Notes: **†** Adjusted for gender, age, hypertension, diabetes mellitus, and chronic kidney disease (CKD) using Spearman’s partial rank correlation analysis. All correlations are statistically significant at ***, P < 0.001

Abbreviations: TC, Total Cholesterol; TG, Triglycerides; HDL-C, High-Density Lipoprotein Cholesterol; LDL-C, Low-Density Lipoprotein Cholesterol; IL-6, Interleukin-6

### Correlation of IL-6 with nutritional indicators in tuberculosis patients

Spearman’s partial rank correlation analysis (non-normal data) was performed to explore the correlations between IL-6 and lipid indicators, both before and after adjusting for five confounders (gender, age, hypertension, diabetes mellitus, and CKD). The results showed that after adjustment, the significant negative correlations of IL-6 with TP (pre-adjustment: *r* =-0.095,* P = 0.011*; post-adjustment: *r* =-0.163,* P = 0.000*) and ALB (pre-adjustment: *r* =-0.520,* P = 0.000;* post-adjustment: *r* =-0.253,* P = 0.000*) persisted; however, the association between IL-6 and GLB changed from statistically significant to non-significant (pre-adjustment: *r* = 0.412,* P = 0.000;* post-adjustment: *r* = 0.044,* P = 0.244*), as illustrated in Table 5.

**Table 5:** Correlation between inflammatory marker IL-6 and nutritional indicators before and after adjustment for confounders (*n* = 724)

Nutritional Indicator	Unadjusted Correlation with IL-6 (*r*)	Adjusted Correlation with IL-6 (*r*)†
TP	–0.095^*^	–0.163^***^
ALB	–0.520^***^	–0.253^***^
GLB	0.412^***^	0.044

Notes: **†** Adjusted for gender, age, hypertension, diabetes mellitus, and chronic kidney disease (CKD) using Spearman’s partial rank correlation analysis. Significance levels are indicated as: ***, P < 0.001, *, P < 0.05

Abbreviations: TP, Total Protein; ALB, Albumin; GLB, Globulin; IL-6, Interleukin-6

### Impact of tuberculosis severity on IL-6-metabolic indicator correlation

To evaluate the impact of tuberculosis severity (sputum smear acid-fast positivity and presence of lung cavities) on the relationships among IL-6, blood lipids and nutritional indicators, we conducted partial correlation analysis. As shown in Table 6, after stratification by sputum smear status and lung cavities, the overall relationships among inflammation, nutrition, and lipid profiles remained largely consistent with those observed before controlling for these variables.

**Table 6:** Correlation among inflammatory marker IL-6, lipid profiles and nutritional indicators in patients (*n* = 724)**†**

Variable	ALB	TC	GLB	HDL-C	LDL-C	TG	TP	IL-6
ALB	-	0.420 ***	-0.400 ***	0.510 ***	0.288 ***	0.130 ***	0.506 ***	-0.408 ***
TC		-	-0.103 ***	0.468 ***	0.888 ***	0.348 ***	0.286 ***	-0.990 ***
GLB			-	-0.266 ***	-0.021	-0.041	0.525 ***	0.107**
HDL-C				-	0.182 ***	-0.229 ***	0.227 ***	-0.470 ***
LDL-C					-	0.341 ***	0.243 ***	-0.878 ***
TG						-	0.084 (0.025)	-0.347 ***
TP							-	-0.276 ***
IL-6								-

Notes: **†** Correlations are partial, controlling for smear (0 = negative, 1 = positive) and cavity (0 = none, 1 = present). Significance levels are indicated as: ***, P < 0.001, **, P < 0.01

Abbreviations: TC, Total Cholesterol; TG, Triglycerides; HDL-C, High-Density Lipoprotein Cholesterol; LDL-C, Low-Density Lipoprotein Cholesterol; TP, Total Protein; ALB, Albumin; GLB, Globulin; IL-6, Interleukin-6

### Determinants of serum IL-6 levels: multiple linear regression analysis

The results of the multiple linear regression analysis identifying independent predictors of serum IL-6 are presented in Table 7. The final model, adjusted for demographic and clinical characteristics, explained 91.3% of the variance in IL-6 levels (adjusted *R²= 0.913, F(10, 712) = 754.883, P < 0.001*). Among lipid parameters, lower levels of LDL-C (*β= -0.600, P < 0.001*), HDL-C *(β= -0.345, P < 0.001*), and TG (*β=-0.158, P < 0.001*) were all independently associated with higher IL-6 concentrations. Regarding nutritional indicators, a lower level of GLB was independently associated with higher IL-6 (*β= -0.041, P = 0.016*), whereas TP showed no significant association. Female gender was also a significant independent predictor of higher IL-6 (*β = 0.214, P < 0.001*).

**Table 7:** Independent predictors of serum IL−6 levels identified by multiple linear regression analysis (*n* = 724)

Predictor	Unstandardized Coefficient (B)	95% Confidence Interval for B	Standardized Coefficient (β)	P-value
**Demographic & Clinical characteristics**				
Gender (Female vs. Male)	0.235	[0.198, 0.272]	0.214	0.000
Age (years)	-0.043	[-0.126,0.040]	-0.013	0.307
Hypertension (Yes vs. No)	0.005	[-0.026,0.036]	0.004	0.763
Diabetes (Yes vs. No)	-0.008	[-0.037,0.021]	-0.006	0.593
CKD (Yes vs. No)	-0.001	[-0.042,0.040]	-0.001	0.949
**Lipid Profiles**				
TG (mmol/L)	-0.382	[-0.445,-0.319]	-0.158	0.000
HDL-C (mmol/L)	-1.222	[-1.331,-1.113]	-0.345	0.000
LDL-C (mmol/L)	-2.206	[-2.322,-2.091]	-0.600	0.000
**Nutritional Indicators**				
TP (g/L)	0.343	[-0.077,0.764]	0.028	0.109
GLB ( g/L)	-0.239	[-0.434,-0.045]	-0.041	0.016
**Model Summary**				
R² / Adjusted R²	0.914 / 0.913			
F-statistic (df)	F(10, 712) = 754.883			
Overall *p-value*	< 0.001			

Abbreviations: IL-6, Interleukin-6; LDL-C, Low-Density Lipoprotein Cholesterol; HDL-C, High-Density Lipoprotein Cholesterol; TG, Triglycerides; TP, Total Protein; GLB, Globulin; CKD, Chronic Kidney Disease

## Discussion

This study clearly reveals significant metabolic and nutritional disorders in patients with active pulmonary tuberculosis using a large sample case-control analysis. These characteristics can be summarized as a metabolic phenotype of “hypolipidemia - hypoalbuminemia - hyperglobulinemia.” Our findings are consistent with other studies that confirm a comprehensive decline in blood lipid levels in tuberculosis patients [10, 11]. Hypolipidemia not only reflects impaired liver synthetic function and depletion of metabolic resources but may also weaken host immune capacity, since cholesterol and its metabolites are key players in immune cell function and membrane structure [12, 13]. Similarly, low levels of TP and ALB are classic markers of malnutrition and disease severity in tuberculosis, linked to higher mortality rates [14]. The elevated levels of GLB are associated with persistent immune activation and inflammatory responses [15]. Our research results are consistent with literature reports, indicating that tuberculosis infection causes systemic metabolic depletion, accompanied by compensatory increases in globulin, which may be related to immune system activation and the synthesis of immunoglobulins.

Partial correlation analysis revealed that after adjusting for five confounding factors (Diabetes, Hypertension, CKD, Age, and Gender), the correlations between lipid indicators and nutritional indicators underwent certain changes. The previously significant correlations of GLB-TC and ALB-LDL-C disappeared, while a significant correlation emerged for GLB-LDL-C. This phenomenon primarily arises because sex hormones (gender) and metabolic disturbances induced by hypertension/diabetes mellitus tend to create “spurious correlations due to synchronous indicator changes” (e.g., GLB-TC and ALB-LDL-C) when unadjusted. Alternatively, age-related metabolic decline or nephropathy-induced lipoprotein-protein excretion disorders may mask true correlations (e.g., GLB-LDL-C). Meanwhile, we observed that after adjusting for the five confounders, no significant correlations existed between TG and any of the three nutritional indicators. These findings underscore that when investigating associations between nutritional and lipid indicators, it is imperative to adjust for demographic and chronic disease confounders to avoid spurious correlations or missed associations, thereby ensuring the authenticity and robustness of conclusions. To further delineate the independent determinants of systemic inflammation, we performed multiple linear regression analysis. After rigorous adjustment for confounders and addressing multicollinearity, the final model identified lower levels of LDL-C, HDL-C, TG, and GLB as significant independent predictors of higher serum IL−6 concentrations (Table 7). This model explained a substantial proportion of the variance in IL−6 levels (adjusted R²= 0.913). Notably, neither TP nor ALB retained independent predictive value in this multivariable context. These results extend our partial correlation findings by confirming that the associations between IL−6 and specific lipid fractions (LDL-C, HDL-C, TG) are robust and independent. Contrary to the suggestion from partial correlations that the IL−6-GLB association might be confounded, regression analysis established GLB as an independent predictor, highlighting a distinct relationship between this protein fraction and inflammation.

The core analytical advance of this study lies in identifying these specific metabolic-nutritional indicators as independent predictors of IL−6 levels. The strong, independent negative associations of LDL-C, HDL-C, and TG with IL−6 strongly support the concept that elevated IL−6 is integrally linked to a dysregulated metabolic state in tuberculosis, suggesting a mechanistic nexus that warrants further longitudinal and experimental validation. Mechanistically, IL−6 may exert its effects through the following pathways: (1) inhibiting de novo synthesis of cholesterol and fatty acids by downregulating the expression and activity of sterol regulatory element-binding proteins (SREBPs) in the liver, leading to hypolipidemia [16]; (2) Interleukin−6 (IL−6) mediates the induction of Perilipin 5 (PLIN5) via the JAK/STAT3 signaling pathway, where PLIN5 serves as a regulator of lipid metabolism [17];(3) promoting skeletal muscle protein breakdown through activation of the ubiquitin-proteasome system and myostatin signaling pathway, resulting in muscle wasting and hypoalbuminemia [18]; (4) simultaneously, IL−6, as a major inducer of the acute phase response in the liver, stimulates the production of inflammatory proteins such as C-reactive protein (CRP), serum amyloid A (SAA), thereby reprogramming the liver’s protein synthesis pattern from maintaining nutrition to supporting inflammation [19]. The independent inverse association with globulin is particularly intriguing and suggests complexity beyond simple acute-phase responses. It may indicate a shift in hepatic synthetic priority away from immunoglobulins during severe inflammation, increased consumption or clearance of specific globulin fractions, or reflect a specific immune phenotype meriting further proteomic investigation.

This “inflammation-metabolism-nutrition” trio has important implications for both clinical practice and public health. First, it explains why tuberculosis patients experience irreversible wasting even when sufficient food is available [20]. Second, malnutrition and metabolic disorders can weaken patients’ tolerance and response to first-line anti-tuberculosis drugs (such as isoniazid and rifampicin), increase the risk of drug-induced liver injury, and potentially lead to treatment failure and relapse [21–23].

The specific lipid and protein fractions identified as independent correlates of IL-6 (LDL-C, HDL-C, TG, GLB) could serve as potential biomarkers for monitoring the metabolic-inflammatory interplay during therapy. From a public health perspective, countries with a high burden of tuberculosis often also face prominent malnutrition issues [24]. Therefore, incorporating nutritional screening (e.g., high-protein, high-energy diets) and potential anti-inflammatory or metabolic-regulatory interventions (such as ω-3 fatty acid and vitamin D supplementation) into routine tuberculosis management may represent a truly cost-effective strategy to optimize treatment outcomes and alleviate disease burden [25–28].

## Study limitations

1. Cross-Sectional Design Constraint: While providing robust cross-sectional insights through simultaneous assessment of inflammation, metabolic, and nutritional markers in a large cohort, the cross-sectional nature inherently limits causal inference. The strong independent associations identified by regression analysis, while controlling for key confounders, cannot establish the direction of causality.
2. Confounder Control Deficiencies: (1) Incomplete documentation of control group socioeconomic status (education/occupation/income) and dietary intake (e.g., absence of 24-hour dietary recall) constrains differentiation of nutritional indicator fluctuations (TP/ALB/GLB) between disease-driven pathological changes and nutrition-related supply disparities. (2) Insufficient characterization of comorbid conditions in controls further limits comprehensive confounder adjustment.
3. IL-6 Measurement Limitation: Dynamic fluctuations of IL-6 with disease progression and acute inflammation render single-admission measurements inadequate for capturing stable cytokine levels, limiting temporal interpretation of its metabolic regulatory effects.
4. Model Interpretation: The exceptionally high explanatory power (R²> 0.9) of the final regression model, although statistically sound, may reflect specific interrelationships within this cohort. This necessitates external validation in independent populations to confirm the generalizability of these specific predictor-inflammation relationships.

## Future research directions

1. Longitudinal Study Design: Prospective longitudinal studies should systematically collect socioeconomic/dietary variables and track the trajectories of the identified biomarkers (IL-6, LDL-C, HDL-C, TG, GLB) during treatment. This would help establish temporality and strengthen causal inference regarding their interrelationships.
2. Mechanistic Validation: Complementary basic experiments are warranted to elucidate molecular pathways underlying IL-6’s metabolic regulation, integrating temporal clinical data to strengthen biological plausibility.
3. Intervention Trials: Informed by these associations, future research should include intervention trials examining whether modulating the identified metabolic-nutritional predictors (e.g., through nutritional supplementation or metabolic modulators) can influence IL-6 levels and, ultimately, clinical outcomes in tuberculosis patients.

## Conclusions

In summary, this study confirms the presence of a “hypolipidemia - hypoalbuminemia - hyperglobulinemia” phenotype in pulmonary tuberculosis patients. Employing multivariable regression analysis, we further identified that lower levels of LDL-C, HDL-C, TG, and GLB are independent predictors of higher IL-6 levels, highlighting a specific inflammatory-metabolic-nutritional nexus. This indicates that IL-6-driven inflammation is a key correlate of this distinct metabolic and protein dysregulation. Our research reinforces the paradigm of tuberculosis as a systemic metabolic disorder. It strongly suggests that comprehensive patient management should look beyond pulmonary symptoms to routinely include assessment of specific lipid profiles and protein fractions. This integrated approach aims to disrupt the vicious cycle of “tuberculosis - inflammation - metabolic wasting - immunodeficiency” and may ultimately contribute to improving global tuberculosis treatment outcomes.

## Supplementary Information

Below is the link to the electronic supplementary material.

Supplementary Material 1

## Data Availability

All the data involved in this study were included in this published article. The original data can be reasonably obtained after consulting the corresponding author by email.

## Abbreviations

IL-6: Interleukin-6
TB: Tuberculosis
TG: Triglycerides
TC: Total Cholesterol
HDL-C: High-Density Lipoprotein Cholesterol
LDL-C: Low-Density Lipoprotein Cholesterol
TP: Total Protein
ALB: Albumin
GLB: Globulin
