# Supplementary material for: Association of serum Interleukin-6 with dysregulated lipid metabolism and nutritional status in patients with pulmonary tuberculosis: a case-control study
Source: BMC Infect Dis. 2026 Feb 13;26:594. doi: 10.1186/s12879-026-12823-8 (PMC13005510; doi:10.1186/s12879-026-12823-8)
Supplement: Supplementary file 1 — Supplementary Material 1 [file 12879_2026_12823_MOESM1_ESM.docx]

| ****Table S1. Comparison of candidate regression models for predictors of serum IL-6 levels**** | | | | | |
| --- | --- | --- | --- | --- | --- |
| Model | Category | Variable Configuration | Key Statistical Findings (in that model) | Adjusted R² | Reason for Selection/Rejection |
| **1** | Lipid Selection | **TC**, HDL-C, TG (excluding LDL-C) | TC significant (p<0.001). HDL-C and TG non-significant. | 0.960 | Rejected. Severe multicollinearity (no individual lipid parameters significant despite high R²). |
| **2** | Lipid Selection | **LDL-C**, HDL-C, TG (excluding TC) | **LDL-C, HDL-C, and TG all significant (p<0.001).** | **0.912** | Selected for lipid component. All three lipid parameters show significant independent associations. |
| **3** | Nutrition Test | TP, **ALB** (excluding GLB) | Neither TP nor ALB was significant (p>0.05). | 0.912 | Rejected. No independent predictive value for ALB in this configuration. |
| **4** | Nutrition Test | TP, **GLB** (excluding ALB) | **GLB significant (p=0.016).** TP non-significant. | **0.913** | Selected for nutrition component. GLB shows independent predictive value when adjusted for TP. |
| **5** | Nutrition Test | **ALB, GLB** (excluding TP) | Neither ALB nor GLB significant (p>0.05). | 0.912 | Rejected. High mutual collinearity likely masked independent effects. |
| **6** | Nutrition Test | **TP, ALB, GLB** (all included) | GLB significant (p=0.030); ALB and TP non-significant; VIF > 20 for all three | 0.913 | Rejected. Severe multicollinearity (VIF > 20) making coefficients uninterpretable. |
| **F** | **Final Model** | **LDL-C, HDL-C, TG, TP, GLB** + All Covariates | **LDL-C, HDL-C, TG, GLB significant.** TP non-significant ; All VIF < 5. | **0.913** | Selected as final model. Combines the robust lipid configuration (M2) and informative nutrition configuration (M4) into a parsimonious, interpretable model without multicollinearity issues. |
| Table S1. This table summarizes the key comparisons during model selection. The lipid model containing LDL-C (Model 2) was preferred over the TC model (Model 1). Among nutritional indicator configurations, only the model containing TP and GLB (Model 4) yielded a significant independent predictor (GLB). The final model (Model F) integrates these choices, representing the most parsimonious and informative configuration. All models included the same demographic and clinical covariates (age, gender, hypertension, diabetes, CKD). | | | | | |
